# Gilberti project and the creation of a linguistic dataset: Conservation and linguistic study of the P'urhépecha language

**DOI:** 10.1016/j.dib.2024.110761

**Published:** 2024-07-17

**Authors:** Agustín Jacinto Zavala, Mauricio González-Avilés, Hermelinda-Servín Campuzano

**Affiliations:** a Centro de Estudios de las Tradiciones, El Colegio de Michoacán, C. Martínez de Navarrete 505 Col. Las Fuentes C.P. 59699 Zamora de Hidalgo Michoacán, Mexico; b Maestría en Ingeniería para la Sostenibilidad Energética, Universidad Intercultural Indígena de Michoacán, carretera Pátzcuaro-Huecorio km. 3 sn C.P. 61614 Pátzcuaro Michoacán, Mexico

**Keywords:** Language preservation, P'urhépecha language, Digital tools, Editable vocabulary, Unpublished texts, Old Spanish

## Abstract

This document provides a dataset transcription and translation of unpublished texts in the P'urhépecha language. The preserved texts are of a religious nature, reflecting the evangelizing efforts of missionaries during the 17th to 19th centuries, with a specific emphasis on the initiatives undertaken by the Gilberti Project at the Center for the Study of Traditions of El Colegio de Michoacán. The investigation introduces innovative digital tools and editable resources, opening new avenues for the study and preservation of the P'urhépecha language, ensuring its relevance and accessibility for future generations.

The Gilberti Project has been active for over two decades, dedicating itself to the analysis of P'urhépecha texts. Beyond its academic role, the project significantly contributes to the conservation and promotion of the p'urhépecha language in several indigenous communities in the state of Michoacán, Mexico, where the language is still alive.

Specifications Table

**Table:** 

Subject	Social Sciences Linguistics
Specific subject area	*Linguistic Documentation: Focuses on the documentation of languages, often with an emphasis on endangered or resource-limited languages. It includes the collection of texts, recordings, and other linguistic data, which is crucial for preserving and studying languages like P'urhépecha.* *Descriptive Linguistics: This branch is dedicated to describing and analyzing the features of a language. The material compiled in the database can be a valuable source for describing grammatical, lexical, and phonetic aspects of P'urhépecha.*
Data format	Raw, Analyzed, Filtered
Type of data	Table
Data collection	*All data are part the “Proyect Gilberti” [Gilberti project] which was organized in 1982 at the Center of the Studies of Traditions of El Colegio de Michoacán, A.C. This project was instituted for Tarasco-speakers graduate students as a graduation option in their Master of Arts, and Ph.D. in Arts at the above-mentioned Center. The project involved the transcription, study and translation of the manuscripts and published books located in diversed repositories in Mexio, the United State and Europe. The dataset, in this work, was organized into four columns: the first and second columns consist of transcriptions of language words or phrases P'urhépecha and old spanish, respectively. The third column offers a direct translation into language contemporary spanish, and the fourth column has been generated using an automatic translator through the use of Google Translator libraries.*
Data source location	The transcriptions are available at the Digital Repository Center of El Colegio de Michoacán in a DVD. The resulting studies and graduation thesis are not includes, as they are available at the Library of El Colegio de Michoacán
Data accessibility	Repository name: PROYECTOGILBERTI Data identification number: DOI: 10.5281/zenodo.10976238 Direct URL to data: https://github.com/PROYECTOGILBERTI/BIGDICTONARY

## 1 Value of the Data

• **Preservation of Cultural Heritage**: The data from the P'urhépecha language are crucial for preserving the linguistic and cultural heritage of the P'urhépecha people in Michoacán. By documenting grammars, dictionaries, and religious texts from the 16th to 19th centuries, these resources help sustain the language and cultural practices, safeguarding them for future generations.
• **Facilitation of Academic Research**: These comprehensive resources serve as a vital tool for researchers in linguistics, anthropology, and history, providing insights into the linguistic features and cultural context of the P'urhépecha language during the Spanish colonial period. The accessibility of these transcribed and translated texts encourages deeper scholarly investigation into this indigenous language.
• **Enhancement of Educational Programs**: The data are invaluable for educational purposes, allowing for the development of curriculum and learning materials focused on the P'urhépecha language and culture. This supports both language revitalization efforts and the education of both native and non-native speakers.
• **Support for Comparative Linguistic Studies**: By providing detailed linguistic data, these resources enable researchers to conduct comparative studies between the P'urhépecha language and other regional or global indigenous languages. Such studies can contribute significantly to the fields of linguistics and indigenous studies, offering broader insights into language structure and evolution.
• **Cultural Enrichment for General Public**: The availability of these resources makes the rich cultural heritage of the P'urhépecha people accessible to a wider audience, promoting a greater understanding and appreciation of indigenous cultures and their historical contexts among the general public.

## 2 Background

The compilation of the P'urhépecha language dataset was driven by the need to preserve and revitalize an endangered indigenous language, integral to the cultural identity of the P'urhépecha community in Michoacán, Mexico. Historically, the language has been underdocumented, with existing resources scattered or inaccessible, posing significant barriers to linguistic and cultural continuity. The Gilberti Project initiated the systematic collection and digitization of historical texts—ranging from grammars and dictionaries to religious manuscripts—dating from the 16th to 19th centuries. This effort aligns with methodological frameworks in linguistic anthropology and digital humanities, which emphasize the importance of preserving linguistic data as both cultural heritage and a means for academic research. The project also responds to a broader theoretical discourse on language endangerment and revitalization, providing essential tools and resources that support linguistic studies and educational initiatives aimed at teaching and learning the P'urhépecha language. The data thus serve as a foundational element for ongoing and future research, enhancing our understanding and sustaining the cultural legacy of the P'urhépecha people.

The data related to the P'urhépecha language, including grammars, dictionaries, religious texts, and more, are invaluable due to their role in preserving an indigenous language and culture. The P'urhépecha language is an essential part of the heritage of the indigenous population in the State of Michoacán, and these documents provide a unique insight into the language, religious practices, and cultural norms of the time. They are also a product of the historical context of the 17th to 19th centuries, reflecting the interplay between indigenous cultures and the Spanish missionaries [1].

## 3 Data Description

**Fig. 1:**
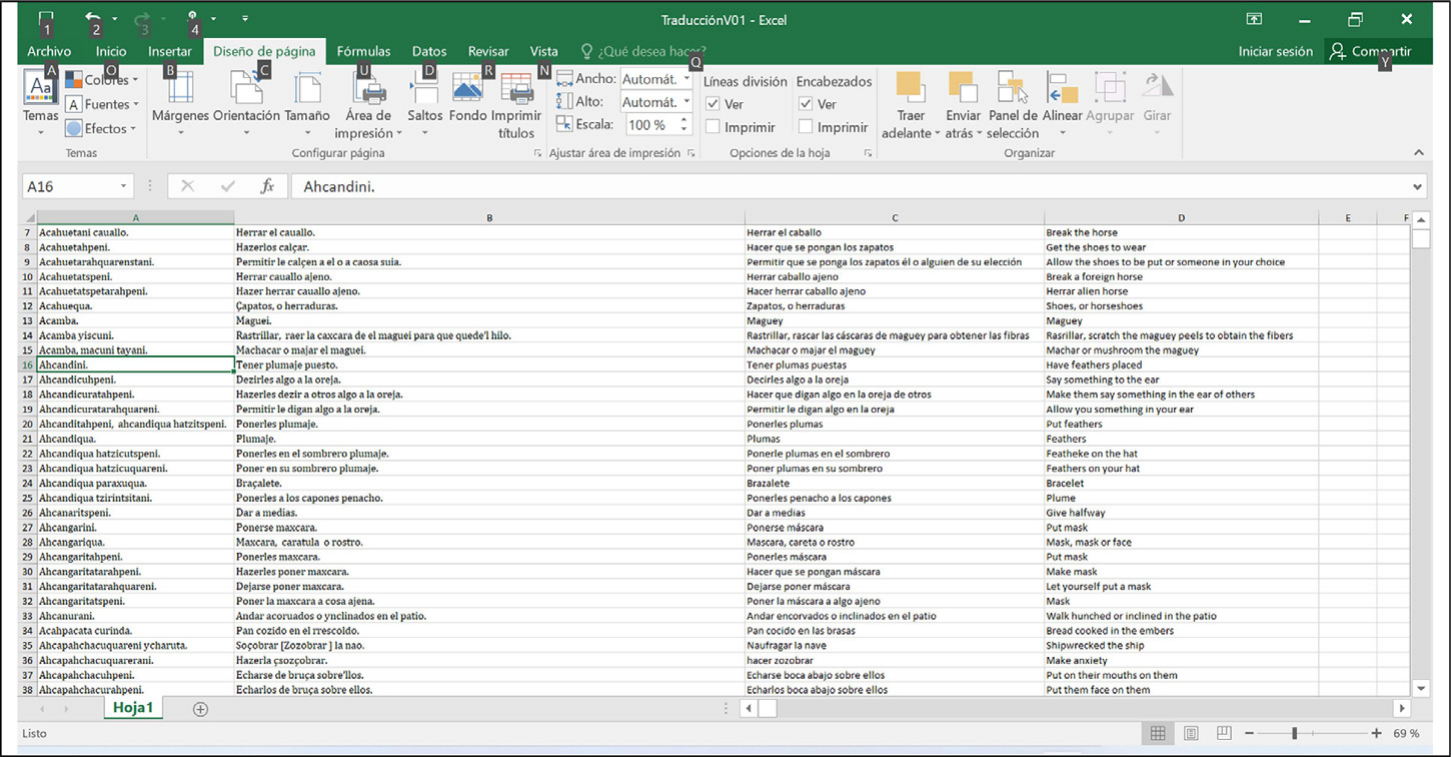
Data structure.

The data are presented in a format of four rows in a spreadsheet file, as shown in Fig. 1:

It is observed that the first column contains words or short phrases in P'urhépecha; the second, their translation into old Spanish; the third, into current Spanish; and the fourth, into American English. The folder named 'DATA' contains the sources from which the data for the spreadsheet were obtained, so most of them published by Dr. Benedict Warren. In addition, it includes some files that are still in the form of images and have not yet been transcribed.

## 4 Experimental Design, Materials and Methods

**Design and Initiation of the Project**: The Gilberti Project began at El Colegio de Michoacán in 1982 when Dr. Benedict Warren made available to the Center for the Study of Traditions copies of microfilms of texts in the Purépecha language that he had obtained from various repositories in the United States. In Michoacán, the first person to start collaborating with him was Father Agustín García Alcaraz, a Purépecha speaker from Naranja, Michoacán, with whom he could consult regarding terminology in this language.

**Objective**: The main goal of this project was to publish the works of Maturino Gilberti (1507–1585) that were written in the language of Michoacán during the 17th century. These writings primarily comprised religious content, but some were also of linguistic nature.

**Program of Activities**: Activities were classified based on the type of material in question:

1. **Paleoimprint (or Paleography) and Edition**: The first activity centered around the paleoimprint (or paleography) and the edition of the works in the language they were originally written, with the majority in the language of Michoacán.
2. **Translation and Publication**: The second activity involved translating the works written in the language of Michoacán and subsequently publishing them.
3. **Relative Studies**: The third activity was based on studies carried out in relation to the edited works.

### 4.1 Text transcription

For Gilberti's texts that were published during his lifetime, the transcription required paleoimprinting techniques based on microfilms. For the microfilms of manuscripts, manual paleography was essential. The initial texts that were transcribed and published include *Grammatica Latina, Arte de la lengua de Mechoacan*, and *Vocabulario en lengua de Mechoacan* [2].

In the context of Jacinto's research, several years were devoted to the manual paleography of manuscripts. This meticulous process served as a foundation upon which new participants could base their work. The initial transcriptions were manually reviewed one or two times by expert paleographers to ensure accuracy. Following these reviews, a native P'urhépecha speaker conducted a manual re-evaluation to validate the reliability of the texts, given the current capabilities of the participants and the nuances of the language.

### 4.2 Translation of the texts

The translations were carefully reviewed and refined manually by native P'urhépecha speakers to ensure they accurately conveyed the original meanings and nuances. The transcription of texts, initiated by Dr. Warren [3], continued at the Center for the Study of Traditions (CET) under the supervision of Dr. Agustín Jacinto Z., and only after several years did the CET decide to open the possibility of pursuing a Ph.D. in Studies of Traditions for Purépecha-speaking students. These students, under the guidance of a tutor, were required to transcribe, study, and translate texts in the Purépecha language available on microfilm in various repositories in Mexico and abroad, especially in the United States, for their thesis work. The transcription, study, and translation work was approved by the CET as an option for obtaining a Master's degree in Studies of Traditions and a Ph.D. in Studies of Traditions. Most of the theses are available at the library of El Colegio de Michoacán.

### 4.3 Materials and tools

The primary materials for this project included microfilms of Gilberti's works, encompassing both published texts and manuscripts. Manual paleography was the primary method used for transcription, supplemented by digital tools for subsequent verification and analysis.

### 4.4 Experimental conditions

Given the nature of the project, the conditions centered on linguistic analysis, manual text transcription, and translation. The experimental conditions were influenced by the available resources, the expertise of the participants, and the inherent challenges of working with historical texts and a unique language. The transcription and review processes were conducted manually by expert paleographers and native P'urhépecha speakers, ensuring a high level of accuracy and reliability.

## Limitations

During the project, several limitations were evident. The transcribing of historical texts, especially those of Gilberti that were published during his lifetime, required a meticulous process of paleoimprint based on microfilms. When dealing with microfilm manuscripts, the challenge was even more pronounced as it demanded intensive paleography. The initial transcriptions often underwent multiple reviews by expert paleographers, indicating potential issues with accuracy and reliability. Additionally, there were challenges associated with the translation of the P'urhépecha language. Despite the availability of dictionaries, the unique construction of words in the P'urhépecha language meant that not all words could be easily found or referenced in existing dictionaries. This was further complicated by the language's innate capacity for creating new words, which made translations even more challenging. This limitation was not entirely addressed even with the publication of comprehensive dictionaries such as the Diccionario Grande de la lengua P'urhépecha. Overall, the inherent complexities of working with ancient texts and the intricacies of the P'urhépecha language posed significant limitations to the project's data collection and curation processes.

## Ethics Statement

The production of the data collected did not involve any human subjects, animal experimentation, nor any data from social media platforms. The authors have read and follow the ethical requirements for publication in Data in Brief.

## CRediT authorship contribution statement

**Agustín Jacinto Zavala:** Project administration, Supervision, Conceptualization, Methodology, Data curation, Resources, Writing – original draft, Investigation, Validation. **Mauricio González-Avilés:** Software, Data curation, Resources, Writing – original draft, Investigation, Visualization. **Hermelinda-Servín Campuzano:** Data curation, Software, Writing – review & editing.

## Data Availability

VOCSBULSRIOVF.xlsx (Reference data) (PROYECTOGILBERTI).
